# Genomic mapping of Suppressor of Hairy-wing binding sites in *Drosophila*

**DOI:** 10.1186/gb-2007-8-8-r167

**Published:** 2007-08-16

**Authors:** Boris Adryan, Gertrud Woerfel, Ian Birch-Machin, Shan Gao, Marie Quick, Lisa Meadows, Steven Russell, Robert White

**Affiliations:** 1 Department of Physiology, Development and Neuroscience, University of Cambridge, Downing Street, Cambridge CB2 3DY, UK; 2 Theoretical and Computational Biology Group, MRC Laboratory of Molecular Biology, Hills Road, Cambridge CB2 0QH, UK; 3 Department of Genetics, University of Cambridge, Downing Street, Cambridge CB2 3EH, UK

## Abstract

**Background:**

Insulator elements are proposed to play a key role in the organization of the regulatory architecture of the genome. In *Drosophila*, one of the best studied is the *gypsy *retrotransposon insulator, which is bound by the Suppressor of Hairy-wing (Su [Hw]) transcriptional regulator. Immunolocalization studies suggest that there are several hundred Su(Hw) sites in the genome, but few of these endogenous Su(Hw) binding sites have been identified.

**Results:**

We used chromatin immunopurification with genomic microarray analysis to identify *in vivo *Su(Hw) binding sites across the 3 megabase Adh region. We find 60 sites, and these enabled the construction of a robust new Su(Hw) binding site consensus. In contrast to the *gypsy *insulator, which contains tightly clustered Su(Hw) binding sites, endogenous sites generally occur as isolated sites. These endogenous sites have three key features. In contrast to most analyses of DNA-binding protein specificity, we find that strong matches to the binding consensus are good predictors of binding site occupancy. Examination of occupancy in different tissues and developmental stages reveals that most Su(Hw) sites, if not all, are constitutively occupied, and these isolated Su(Hw) sites are generally highly conserved. Analysis of transcript levels in *su*(*Hw*) mutants indicate widespread and general changes in gene expression. Importantly, the vast majority of genes with altered expression are not associated with clustering of Su(Hw) binding sites, emphasizing the functional relevance of isolated sites.

**Conclusion:**

Taken together, our *in vivo *binding and gene expression data support a role for the Su(Hw) protein in maintaining a constant genomic architecture.

## Abstract 2

An analysis of *Drosophila *Su(Hw) binding allowed the identification of new, isolated, binding sites, and the construction of a new binding site consensus. Together with gene expression data, this supports a role for Su(Hw) in maintaining a constant genomic architecture.

## Background

Insulator elements are proposed to play a key role in the organization of transcriptional regulation within the eukaryotic genome [1,2]. They were first identified as DNA sequences that regulate interactions between promoter and enhancer elements, and are operationally defined as sites that, when positioned between an enhancer and a promoter, block this enhancer/promoter interaction while still allowing the enhancer to operate on other promoters. This function suggests that insulators act to organize independent gene regulatory domains in the genome by preventing inappropriate enhancer/promoter interactions. In *Drosophila*, several insulator elements have been identified, for example the *Fab-7 *insulator in the bithorax complex [3], the *scs *and *scs' *insulators flanking the *hsp70 *locus at 87A7 [4], and the *gypsy *insulator [5]. One of the best characterized of these is the *gypsy *insulator, a 340 base pair (bp) element located within the 5'-untranslated region of the *gypsy *transposable element. The *gypsy *insulator contains 12 binding sites for the zinc finger protein Suppressor of Hairy-wing (Su [Hw]) [6], and Su(Hw) is required for insulator function. In addition to Su(Hw), the *gypsy *insulator complex also includes the BTB/POZ domain proteins Mod(mdg4) 2.2 [7,8] and Centrosomal Protein 190 [9], together with dTopors (a ubiquitin ligase) [10].

Although their mechanism of action remains unresolved, insulators have several properties that indicate a key role in the organization of transcriptional regulation. In vertebrates, almost all characterized insulator elements are associated with the binding of the zinc finger protein CCCTC-binding factor (CTCF), and important roles for these elements have been proposed in gene regulation, in the organization of transcriptional domains, and in imprinting [11,12]. Insulators can protect transgenes from position effects, suggesting a potential role in the separation of domains of differing chromatin state [2]. A CTCF site maps to a chromosomal domain boundary at the mouse and human c-*myc *gene [13], and CTCF sites mark boundaries of chromatin states at the chicken β-globin gene [14]. Furthermore, there is evidence that insulators organize the genome into loops that may represent independent regulatory domains, and it has been proposed that insulators may form the bases of such loops [15,16]. In addition, the Su(Hw) protein is located in a punctate pattern at the nuclear periphery [17] and genetic screens in yeast have identified a prominent role for the nuclear pore in insulator function, potentially as a site for the tethering of chromosomal loops. Thus, insulators are proposed to play a key role in the organization of chromatin within the nucleus by being tethered to nuclear structures [18].

Immunolocalization of Su(Hw) on the polytene chromosomes of *Drosophila *salivary glands indicates binding of Su(Hw) at several hundred sites in the genome [19]. These sites are presumed to represent endogenous insulators; however, until recently, the only characterized *in vivo *Su(Hw) target was the *gypsy *transposable element, and this has been the paradigm for Su(Hw) function for many years. Recently, two groups independently identified an endogenous genomic Su(Hw) insulator, 1A-2, separating the *yellow *gene from the achaete-scute complex [20,21]. A 454 bp fragment containing two binding sites for Su(Hw) was demonstrated to provide *in vivo *enhancer blocking activity in a transgenic insulator assay. The absence of a dense cluster of Su(Hw) binding sites suggested that endogenous Su(Hw) insulators may differ from the *gypsy *paradigm. More recently, an *in vitro *strategy identified potential new endogenous binding sites and confirmed that clustering of binding sites is not a requirement for insulator function. Single binding sites were shown to be capable of mediating strong insulation [22]. An *in silico *approach has also been used to predict endogenous Su(Hw) binding sites [23]. Testing of these candidate sites in an enhancer blocking assay supports the functional relevance of single and double sites. Clearly, the identification of *in vivo *endogenous Su(Hw) target sites is an important goal in our efforts to elucidate the nature of Su(Hw) insulators and in the investigation of their role in the organization of transcriptional regulation at the genomic level.

In this report we present the characterization of *in vivo *Su(Hw) binding sites across a 3 megabase (Mb) region of the *Drosophila *genome. Taking the *Adh *region from *kuzbanian *to *cactus *on chromosome 2L as a representative genomic region, we have identified approximately 60 Su(Hw) binding sites using chromatin immunopurification in concert with genomic microarrays (chromatin immunopurification [ChIP]-array). These sites reveal a robust binding site consensus sequence and enable analysis of genomic context, developmental occupancy, and conservation and function of Su(Hw) binding sites.

We introduce a new approach here - a ChIP strategy that uses anti-green fluorescent protein (GFP) antiserum to immunopurifiy chromatin from a fly strain carrying a GFP-tagged Su(Hw) fusion protein. This approach is attractive as a general strategy for mapping transcription factors in *Drosophila *because it will enable the use of a well characterized antiserum for immunopurification, avoiding the complications of variable properties and availability of antisera specific for individual transcription factors/DNA binding proteins. Combining our approach with ongoing efforts to generate a library of GFP tagged proteins *via *transposon mediated exon insertion [24] provides a strategy for large-scale investigation of protein-DNA interactions in *Drosophila*.

## Results

### Identification of Su(Hw) *in vivo *binding locations

We have used ChIP-array to investigate the *in vivo *binding of the Su(Hw) protein in a representative genomic region; the 3 Mb *Adh *region [25]. This is a well characterized region of chromosome 2L containing the chromosomal stretch from *kuzbanian *to *cactus*. It encompasses approximately 250 genes, or 2.5% of the *Drosophila *euchromatic genome. The *Adh *region is represented on our microarrays as a 1 kilobase (kb) genomic tile path. The full array design for the *Adh *region is described in the report by Birch-Machin and coworkers [26] and the array has been supplemented with other selected *Drosophila *genomic sequences; of particular relevance here is a 1 kb genomic tile covering 130 kb of the *achaete-scute *complex.

For the ChIP-array, we generated chromatin fragments from a *Drosophila *strain expressing a Su(Hw)-GFP fusion protein and used anti-GFP antibody for immunopurification. This approach has the advantage that it offers a generalized strategy for the localization of chromatin-associated proteins in *Drosophila *using a common, well characterized antibody for immunopurification. The Su(Hw)-GPF transgenic line expresses the fusion protein under the regulation of *su*(*Hw*) control elements in a genetic background that is deleted for the *su*(*Hw*) gene [17]. In this strain, the Su(Hw)-GFP rescues the female sterility phenotype of the *su*(*Hw*) mutation. We assessed the immunopurifications by standard polymerase chain reaction (PCR) assays using specific primer pairs and could demonstrate clear enrichment for known Su(Hw) targets, the *gypsy *insulator, and the 1A-2 site in the *achaete-scute *region [20,21], but no enrichment for a *Gpdh *control fragment (data not shown). For the microarray analysis, the immunopurified DNA resulting from the specific (rabbit anti-GFP) ChIP was compared with DNA from control immunopurifications performed from the same chromatin (using normal rabbit serum). Purified DNA was amplified by ligation mediated PCR and labelled with a fluorescent dye. Technical replicates with dye swap labeling were used to control for dye incorporation bias. After hybridization to the array, scanning, and variance stabilization normalization (VSN) [27], enrichment was determined by Cy3/Cy5 ratio.

Su(Hw) is ubiquitously expressed and is proposed to play a general role in the organization of transcriptional regulation; however, it is not known whether this organization is tissue specific. To obtain a view of Su(Hw) binding in different tissues at different stages of development, three sources of chromatin were examined: 0 to 20 hour embryos, third instar larval brain, and third instar larval wing imaginal disc. For each chromatin source four biological replicates (independent chromatin preparations) were used and the data were combined into averages of biological replicates using CyberT [28]. Raw microarray data are available from the National Center for Biotechnology Information Gene Expression Omnibus site [29] as GSE4691 and summarized in Additional data file 1.

**Figure 1:**
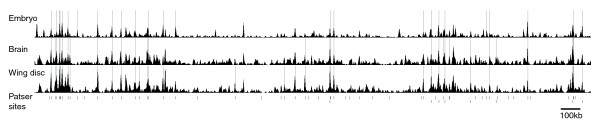
Su(Hw) binding profile across 3 Mb *Adh *region. Schematic of enrichment profiles for embryo, brain, and wing imaginal disc are shown as a plot of enrichment of array fragments against genomic coordinates. Light gray vertical lines on the plots indicate fragments with enrichment greater than 1.7-fold. The positions of high scoring Patser matches to the new Suppressor of Hairy-wing (Su [Hw]) binding consensus are indicated below the enrichment plots. The upper line indicates positions of matches with *P *< e^-15^, and the lower line indicates positions of matches with *P *between e^-12 ^and e^-15 ^and having enrichment >1.7-fold in at least one of the chromatin sources. Annotation tracks are provided in Additional data file 9. kb, kilobases; Mb, megabases.

To generate a list of genomic fragments associated with Su(Hw) binding, we selected fragments exhibiting a mean enrichment above 1.7-fold in the Su(Hw)-GFP data from any one of the three chromatin sources. Pruning this list to remove eight fragments with single extreme outlier values (identified by a CyberT t-value < 1) results in 105 candidate Su(Hw) binding fragments in the *Adh *region. The map of these sequences across the *Adh *region is presented in Figure 1.

**Figure 2:**
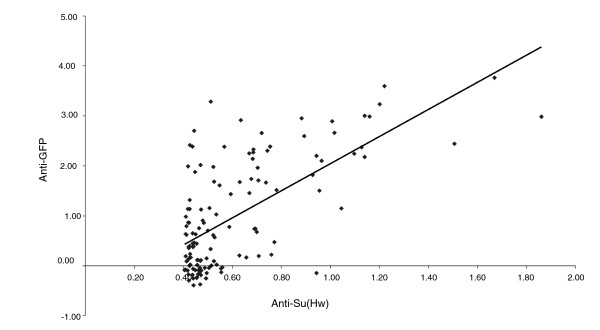
Correlation of ChIP enrichment using either anti-Su(Hw) on wild-type chromatin or anti-GFP on chromatin from Su(Hw)-GFP transgenic. The enrichment values are plotted as the arsinh transformation (approximately equivalent to the log2 scale) of the ratio of specific versus control ChIP. Correlation coefficient is 0.66. ChIP, chromatin immunoprecipitation; GFP, green fluorescent protein; Su(Hw), Suppressor of Hairy-wing.

The dataset was validated using three approaches. First, we examined the array data for known targets. Although the *gypsy *transposable element is not represented on the array, the genomic tile from the *achaete-scute *region covers the 1A-2 Su(Hw) site, which serves as an internal control, and the corresponding array fragment (as-c.1) exhibited clear enrichment. For example, for the dataset derived from embryonic chromatin, the mean fold enrichment is 1.8 with *P *= 7 × 10^-3^. Second, we selected a few fragments over the enrichment range and tested their enrichment employing specific PCR following ChIP using wild-type *Drosophila *chromatin and anti-Su(Hw) antiserum. All fragments showed appropriate ChIP enrichment (data not shown). Third, the DNA from ChIP using anti-Su(Hw) antiserum was labeled and hybridized to the array to generate an array dataset for comparison with the anti-GFP dataset. The two datasets are compared in Figure 2 and show good correlation.

### An improved Su(Hw) binding consensus

**Figure 3:**
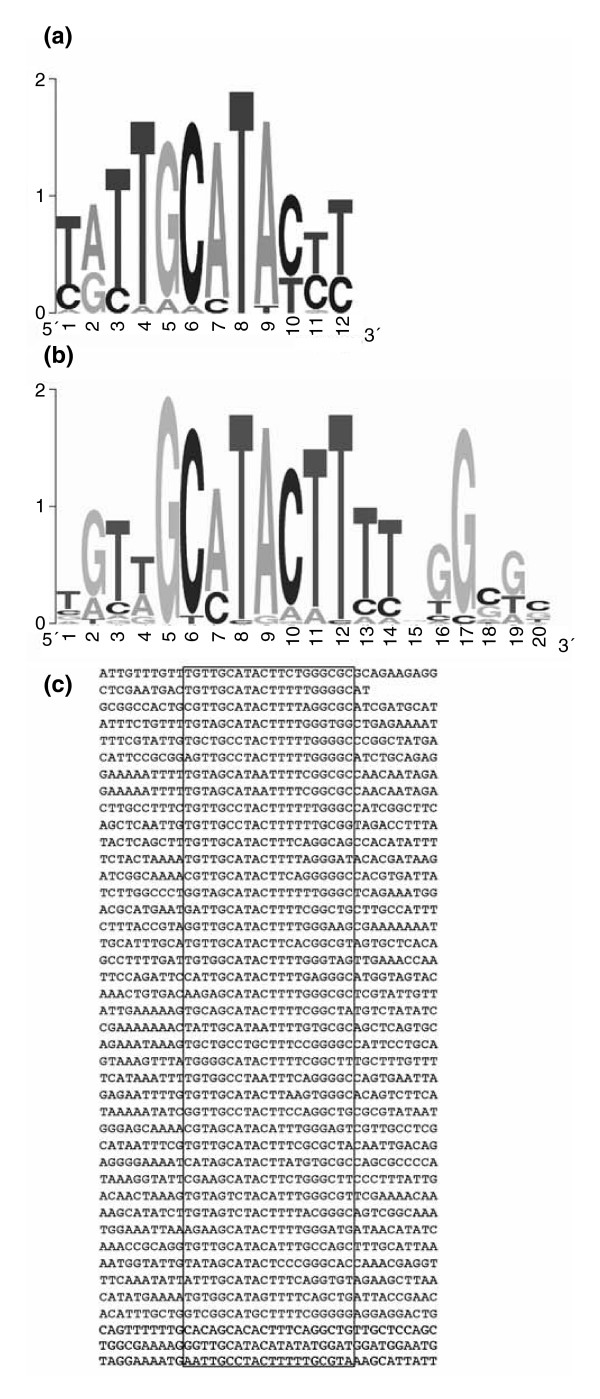
Enhanced Su(Hw) binding site consensus derived from *in vivo *ChIP. **(a) **WebLogo of the *gypsy *consensus. **(b) **WebLogo of the new consensus. **(c) **Aligned stack of the motif identified by MEME; 42 sites contained in 41 array fragments. The box indicates the 20 base pair sequences corresponding to the WebLogo in panel b. ChIP, chromatin immunopurification; Su(Hw), Suppressor of Hairy-wing.

To identify potential Su(Hw) binding sites within enriched fragments, the top binding candidates were submitted to the MEME motif discovery tool [30], to search for potential binding motifs. Because MEME accepts up to 60 kb, the top 63 fragments from the list of 105 candidate binding fragments were submitted. The top motif found by MEME (e-value = 1.3 × 10^-73^) is present in 41 out of the 63 fragments and has the consensus TGT(TA)GC(AC)TACTTTT(GAC)GG(CG)GT)(CG). This is clearly related to both the characterized 12 bp Su(Hw) binding consensus, namely (TC)(AG)(TC)TGCATA(CT)(TC)(TC), derived from the Su(Hw) binding motifs in the *gypsy *transposon [31] (Figure 3a) and the (TC)(TA)GC(AC)TACTT(TAC)(TC) consensus derived from a recent *in vitro *analysis [22]. The sequence matches and the derived WebLogo are presented in Figure 3, and the strength of this consensus clearly indicates the identification of genuine *in vivo *Su(Hw) binding sites.

It is interesting to compare our set of endogenous Su(Hw) sites with the *gypsy *insulator. The 340 bp *gypsy *insulator contains a cluster of 12 Su(Hw) binding sites that share a (TC)(AG)(TC)TGCATA(CT)(TC)(TC) consensus embedded in AT-rich sequences. The new Su(Hw) sites revealed by ChIP array show several differences from the *gypsy *sites. First, unlike the *gypsy *insulator, the endogenous binding sites are not tightly clustered; 40 out of the 41 enriched fragments have a single match to the consensus and only one fragment contains two matches. Second, the binding sequence we derive does not conform to the model of a conserved consensus flanked by AT-rich sequences [31,32]. The sequences flanking the positions corresponding to the 12 bp *gypsy *consensus are not consistently AT rich, although there is a conserved run of four Ts starting at the position corresponding to the 11th bp of the *gypsy *consensus. The T at position 4 in the *gypsy *consensus is noticeably less conserved than the other positions and strong conservation, particularly of the G at position 17, extends beyond the run of Ts at positions 11 to 14. Significantly, the highly conserved bases at positions 2(G), 5(G), 6(C), 10(C), and 17(G) are in excellent agreement with the positions of G residues determined as contact residues in methylation interference experiments with Su(Hw) binding to a single site from the *gypsy *insulator [32]. This observation further strengthens our conclusion that we have successfully identified the *in vivo *Su(Hw) binding sites.

We were interested in determining whether the ChIP enriched fragments showed any other conserved sequences in addition to the Su(Hw) sites that might reveal other DNA binding activities associated with insulator sequences. The MEME results do reveal a CA repeat that is present in 42% of the fragments containing a Su(Hw) motif (e-value = 2.8 × 10^-23^) and in most cases the repeat occurs within 100 to 200 bp of the Su(Hw) motif. However, an alternative tool for motif finding, namely NestedMICA [33], which is generally more resistant to low complexity artefacts, identified the Su(Hw) consensus but not the CA repeats as enriched motifs. Thus, the significance of these CA repeats cannot be assessed at present.

### Correlation between sequence matches to Su(Hw) binding consensus and binding data

The identification of a new expanded Su(Hw) binding consensus allowed us to investigate the link between DNA sequence and the *in vivo *occupancy of predicted Su(Hw) binding sites. We used the 42 occurrences of the pattern identified by MEME within the set of enriched fragments to build a position-specific weight matrix (Additional data file 2). The Patser profile matching tool [34] was then used to search for matches within the 3 Mb of genomic sequences on the microarray. The full Patser data are provided in Additional data file 3. In summary, if we consider the 20 most enriched fragments, ordered by average enrichment in all three chromatin sources, then we see a striking match to high scoring Patser consensus sequence hits (Table 1). All of these highly enriched fragments exhibit good Patser scores with the exception of four fragments; three of these (ADH-690, ADH-3001 [ADH-1199], and ADH-2585) are neighbours to highly enriched fragments that do contain high scoring Patser sites.

**Table 1:** The top 20 fragments

Fragment ID	Fragment ID Sequence	Patser		Enrichment in			Mean
			
		Score	*ln*(*P*)	Embryo	Brain	Wing disc	
ADH-3002(ADH-1200)	faaatGTTGCATACTTTTAGGGATAcacg	16.75	-19.14	2.23	2.35	2.78	2.45
ADH-1585	ftaaaGAAGCATACTTTTGGGATGAtaac	14.14	-16.32	1.87	1.65	2.37	1.96
ADH-2189	faccaTGCCCTCAAAAGTATGCAATggaa	16.15	-18.43	2.06	1.99	1.53	1.86
ADH-2945(ADH-480)	fgacaAGAGCATACTTTTGGGCGCTcgta	16.19	-18.47	1.71	1.43	2.08	1.74
ADH-1112	ftgctTTACGCAAAAAGTAGGCAATtcat	10.66	-13.35	1.66	1.56	1.81	1.68
ADH-454	fttatGGGGCATACTTTTCGGCTTTgctt	14.08	-16.27	1.33	1.49	2.19	1.67
ADH-336	fgtctACCGCAAAAAAGTAGGCAACacaa	16.33	-18.63	1.65	1.34	2.03	1.67
ADH-2586	fttgtGTTGCATACTTAAGTGGGCAcagt	14.51	-16.68	1.46	1.82	1.60	1.63
ADH-178	fttgtGCTGCCTACTTTTTGGGGCCcggc	18.03	-20.82	1.38	1.38	1.99	1.58
ADH-150	fttttGTAGCATAATTTTCGGCGCCaaca	18.09	-20.92	1.41	1.21	2.01	1.54
ADH-125	fcggaGTTGCCTACTTTTTGGGGCAtctg	18.89	-22.13	1.02	1.81	1.79	1.54
ADH-690*	fgctcGTTGCCGCCATTACTGCTGTttgt	1.36	-7.69	0.78	1.28	2.35	1.47
ADH-3001(ADH-1199)*	faatcGTAGCCTAAAATTATGGTAAgatt	3.58	-8.83	0.76	1.66	1.99	1.47
ADH-2808	fno Patser hit			1.00	1.34	2.01	1.45
ADH-2101	fattaTTTGCATACTTTCAGGTGTAgaag	12.67	-14.98	1.33	1.15	1.81	1.43
ADH-96	fttcgAACGCCCAAATGTAGACTACactt	12.77	-15.06	0.93	1.44	1.81	1.39
ADH-405	fttcaACTACCCAAAAGTATGCCACaatc	15.02	-17.19	1.62	1.31	1.21	1.38
ADH-141	fttttGTAGCATAATTTTCGGCGCCaaca	18.09	-20.92	1.18	0.99	1.72	1.30
ADH-1563	fctccTCCCCCGAAAAGCATGCCGAccag	11.59	-14.07	0.74	1.39	1.75	1.29
ADH-2585*	fctccACTGCCCAGAAATTTGCAATtata	5.14	-9.69	1.34	1.51	0.97	1.27

Enrichment is arsinh transformation (approximately equal to log_2 _ratio). Fragments marked with an asterisk are neighbours to fragments with high scoring Patser hits (*P *< e^-15^).

**Figure 4:**
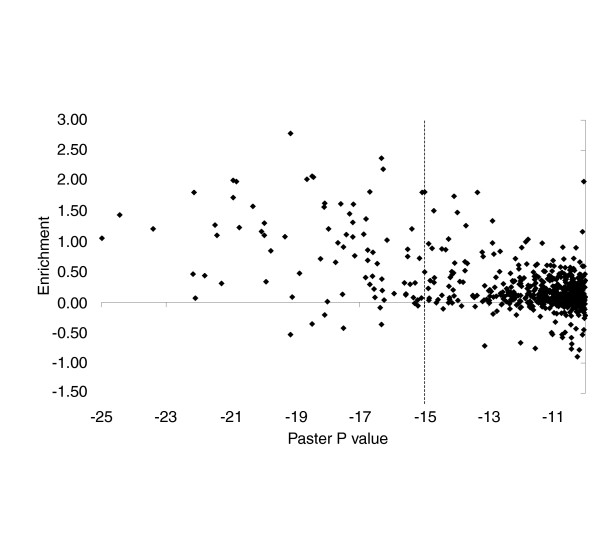
Closeness of match to the Su(Hw) binding site consensus is associated with *in vivo *binding. The Patser *P *value for each Patser match is plotted against the enrichment (arsinh transformation; approximately equal to log_2 _ratio) of the fragment containing the matching sequence. The enrichment value is the highest mean value from the three chromatin sources. The vertical line indicates the Patser *P *= e^-15^; for matches with *P *< e^-15^, 63% show enrichment greater than 0.5 (1.4-fold) and 53% show enrichment greater than 0.8 (1.7-fold). Su(Hw), Suppressor of Hairy-wing.

From a plot of ChIP enrichment versus Patser *P *value, it is clear that closeness of Patser match is correlated with fragment enrichment in the ChIP experiments (Figure 4). Of the Patser hits with a *P *value better than e^-15^, 63% show enrichment greater than 1.4-fold and 53% show enrichment greater than 1.7-fold. Thus, the occurrence of a Patser hit with a *P *value better than e^-15 ^is a strong predictor of *in vivo *occupancy in at least one of the chromatin sources. Additional validation is presented in Additional data file 4, in which we show that seven out of eight of the Patser predicted sites we tested outside the *Adh *region are indeed occupied by Su(Hw) *in vivo*.

This relationship can be seen in Figure 1, in which both the high scoring Patser hits and the ChIP enriched fragments are mapped across the *Adh *region. The plot demonstrates a clear concordance between high scoring Patser hits and ChIP-array enrichment. If we take the Patser sites that have a *P *value less than e^-12 ^and that lie within fragments that show an enrichment of more than 1.7-fold in the ChIP-array, we identify 60 sites of Su(Hw) binding within the 3 Mb *Adh *genomic region.

**Figure 5:**
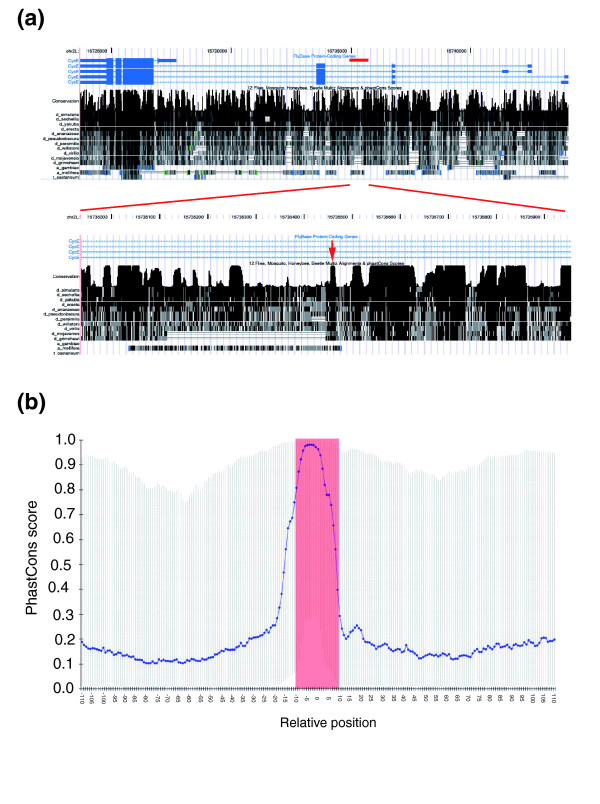
Conservation of Su(Hw)and Su(Hw) binding sites. **(a) **Example of a conserved Suppressor of Hairy-wing (Su [Hw]) binding site in an intron of the *cyclin E *gene. Although the overall conservation of the intron is variable, the binding site itself is a conserved entity. **(b) **PhastCons scores across all 2,281 predicted genomic Su(Hw) binding sites with a Patser *P *value < e^-15^. The binding sites are centred over position 0 and 100 base pairs left and right of the site are shown. The blue line indicates the median PhastCons score for a given position, and the black bar shows the 25th and 75th percentiles of the scores. It is evident that Su(Hw) binding sites are generally highly conserved, whereas their genomic context is not.

We examined the conservation of the identified Su(Hw) binding sites, comparing *Drosophila melanogaster *with available sequences from other *Drosophila *spp. and other sequenced insects, namely the mosquito *Anopheles gambiae*, the honey bee *Apis mellifera*, and the beetle *Tribolium castaneum *(Figure 5). The analysis indicates that the *D. melanogaster *Su(Hw) binding sites are well conserved within the drosophilids; even when located in generally less conserved genomic contexts such as intergenic or intronic sequences, Su(Hw) binding sites stand out as conserved islands (Figure 5a). However, there is little evidence of site conservation in the syntenic regions from the other insects. Within the drosophilids, binding site conservation provides a test of functional relevance, and we find that a good match to the consensus (represented by Patser *P *value) is associated with greater conservation (data not shown). Importantly, binding site conservation is consistent for all Patser predicted binding sites throughout the fly genome (Figure 5b).

Protein homology searches indicate clear Su(Hw) orthologs within drosophilid species (data not shown), but they suggest that although both *Apis *and *Anopheles *contain related zinc finger proteins, they lack clear Su(Hw) orthologs. Together with the lack of binding site conservation, this suggests that Su(Hw) is a species restricted protein; this is in contrast to other insulator associated molecules such as CTCF, which is conserved at least from fly to human [35,36].

### Are Su(Hw) binding sites always occupied?

We looked at the *in vivo *Su(Hw) binding profile in chromatin extracted from three different *Drosophila *tissues, namely embryo, wing imaginal disc, and larval brain, to explore the issue of whether Su(Hw) binding is developmentally regulated or constitutive. As illustrated in Figure 1, the binding profiles of Su(Hw) are very similar in the three chromatin sources examined. If we look at the mean enrichment values for the top 20 enriched fragments, all 20 show greater than 1.6-fold enrichment in all three chromatin sources, and of the top 50 all show greater than 1.4-fold enrichment in all three sources. At the level of individual fragments, we identified a few fragments that show relatively strong enrichment in chromatin from one or two of the sources and little or no enrichment in chromatin from the third source (for instance, Adh-34). To test whether these values represent genuine tissue specific Su(Hw) binding or simply occasional false negatives expected in a microarray based approach, we analyzed a selection of such cases using PCR assays with specific primers. This analysis failed to replicate the selective lack of enrichment from a particular tissue (data not shown). In summary, we find no convincing evidence for tissue specific binding and conclude that most, if not all, Su(Hw) sites are constitutively occupied.

### Genomic environment of the Su(Hw) binding sites

**Figure 6:**
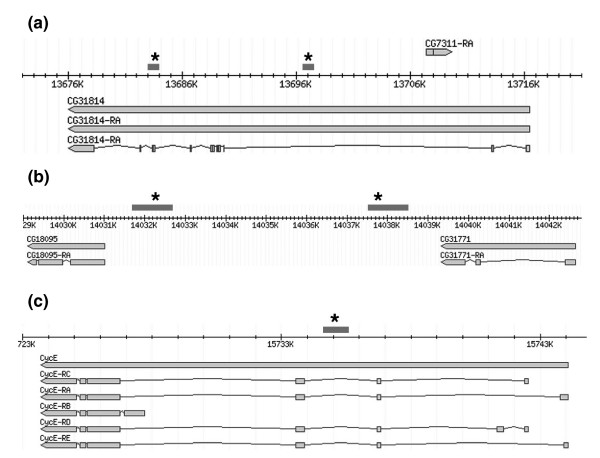
Selected genomic Su(Hw) binding sites. **(a) **Intronic sites in CG31814. **(b) **Sites separating genes transcribed from the same strand (CG18095 and CG31771). **(c) **Suppressor of Hairy-wing (Su [Hw]) site in the *cyclin E *(CycE) gene. Gene models are from the FlyBase genome browser [55]; dark gray bars represent enriched 1 kilobase fragments from the tiling array and asterisks represent the location of Patser sites.

Identification of 60 Su(Hw) binding sites within the 3 Mb *Adh *region enabled us to investigate the relationship between Su(Hw) binding sites and annotated genome features. Our starting point was the simple view that a protein predicted to play a key role in the regulatory architecture of the genome and to insulate separate regulatory domains might identify a particular genomic context; for example, insulator sites might be positioned well away from transcription units. However, we find that the data do not support this; although most of the sites we identified in the *Adh *region are intergenic (63%), this leaves a considerable number that map within transcription units. Intergenic sites are found both between tandem and opposite strand transcription units with no clear preference. Of the intragenic sites, none are located within coding regions; 88% map within introns and the remainder are located in 5'-untranslated regions. Figure 6 shows examples of Su(Hw) binding site locations in association with transcription units. Few of the sites we have identified map to regions in which regulatory elements have been well characterized. One of the few genes in the *Adh *region where the enhancer structure has been studied is the *cyclin E *gene [37]. A complex set of tissue specific regulatory elements that overlap a maternal transcript lying upstream of the zygotic transcription start has been identified. A Su(Hw) binding site is located within the second intron of the maternal transcript and several kilobases upstream from the zygotic transcription unit (Figure 6c). It lies within an enhancer that regulates several tissue specific components of *cyclin E *gene expression, where it would be potentially capable of insulating the promoter from characterized distal enhancers.

**Figure 7:**
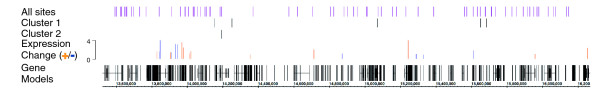
Expression changes in the 3 Mb *Adh *region with respect to Su(Hw) binding sites. Expression changes (as absolute fold change according to the scale bar) are indicated by the bars above the gene models, with upregulated genes in orange and downregulated genes in blue. The bars mark the 5' end of each gene. The location of Su(Hw) binding sites are plotted on the three rows All sites, Cluster 1, and Cluster 2. Cluster 2 indicates the two sites within 100 base pairs of each other. Cluster 1 indicates the six pairs of sites within 1 kilobase of each other. All sites plots the locations of the remaining 83 sites in the region. The maps are plotted and rendered using the Affymetrix Integrated Genome Browser.

We also analyzed the clustering of Su(Hw) sites in the *Adh *region because the *gypsy *insulator contains tightly clustered sites and previous studies have suggested a requirement for multiple sites for maximal insulator function [31]. Of the Patser hits with a *P *< e^-15^, only two pairs of sites are separated by less than 300 bp and only six pairs of sites are separated by less than 1 kb (Figure 7). We conclude that the majority of Su(Hw) sites occupied in the genome are present as single sites and that clustering of multiple sites is not required for Su(Hw) localization on chromatin.

### Su(Hw) sites and DNA bendability

**Figure 8:**
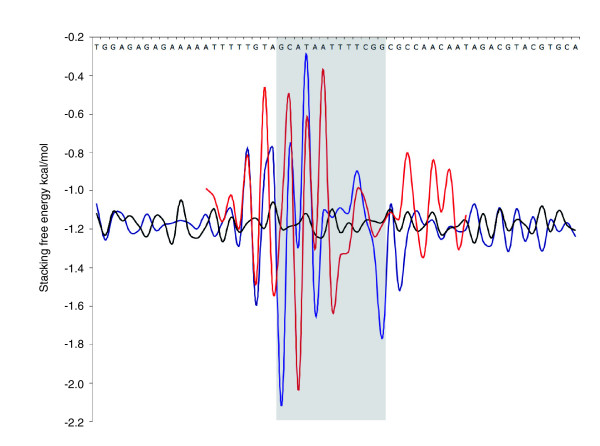
The Su(Hw) binding site has a pronounced DNA flexibility profile. Higher stacking free energy values are associated with DNA flexibility [38]. Blue indicates the stacking free energy profile for 100 best matches to Suppressor of Hairy-wing (Su [Hw]) consensus based on Patser *P *value; black indicates the profile for 100 random sequences; and red indicates the profile for the 12 Su(Hw) sites in the *gypsy *element. A representative sequence is given at the top. The gray zone marks the region between the highly conserved G nucleotides at positions 5 and 17 in the new Su(Hw) binding consensus.

In 1990 Spana and Corces [32] found that local DNA conformation plays a role in the specificity of the interaction between Su(Hw) and its binding sites in the *gypsy *insulator. Their analysis indicated that the AT-rich sequences flanking the core Su(Hw) binding sites were sites of DNA bending, and mutations that interfered with DNA bending reduced *in vivo *insulator activity. Because the endogenous *in vivo *binding sites that we identify here do not obviously conform to the core plus flanking AT-rich sequence arrangement of the *gypsy *insulator sequences, we examined the biophysical characteristics of these sites to characterize their bendability profiles. We used the DNA stability parameters defined by Protozanova and coworkers [38] to provide a measure of DNA flexibility and, as shown in Figure 8, our endogenous Su(Hw) sites exhibit a strong biophysical signature. The strikingly symmetrical profile reveals two stiff elements (centred on the highly conserved G residues at positions 5 and 17), which flank more flexible sequences. The R bend sequence identified by Spana and Corces [32] is conserved as a run of Ts from positions 11 to 14 and forms part of the flexible region. Interestingly, the averaged profile across the 12 *gypsy *element sites differs from the profile across our endogenous sites; although the *gypsy *sites have the left-hand stiff element, they lack the right-hand flexibility minimum.

### Gene expression changes in Su(Hw) mutants

In transgenic insulator assays, the activity of the gypsy insulator is abolished in *su*(*Hw*) mutants, indicating that Su(Hw) is required for insulator function. However, for the endogenous genome, the consequences of loss of Su(Hw) are less obvious because mutant flies are viable and exhibit no clear abnormalities except for female infertility.

Recently, Parnell and coworkers [23] showed, using reverse transcription PCR, that a few genes close to putative endogenous Su(Hw) binding sites, selected on the basis of site clustering, have expression changes in *su*(*Hw*) mutants. To extend this analysis and to relate gene expression to our newly identified endogenous Su(Hw) binding sites, we carried out a genome-wide survey of transcription levels in *Su*(*Hw*) null mutants using whole-transcriptome microarrays. We analyzed RNA extracted from both whole third instar larvae (synchronized during the short time when they are soft white pre-pupae) and wing imaginal discs dissected from similarly staged animals. RNA was prepared from larvae of the genotype *su*(*Hw*)^*v*^, *P *[*CaS X*/*K5.3*]/*Df*(*3R*)*ED5644*, which is a *su*(*Hw*)-null background, and from the heterozygotes *su*(*Hw*)^*v*^, *P *[*CaS X*/*K5.3*]/*Or *and *Df*(*3R*)*ED5644*/*Or*, in order to control for genetic background. For each genotype, four independent biological replicates were prepared and co-hybridized with a pool of RNA extracted from similarly staged wild-type larvae. After hybridization and scanning, array data were normalized with VSN and significant changes in gene expression determined using CyberT [28]. In both whole animal and wing disc experiments, we observed a fivefold to sevenfold decrease in *su*(*Hw*) expression, a positive control for the behavior of the arrays.

**Figure 9:**
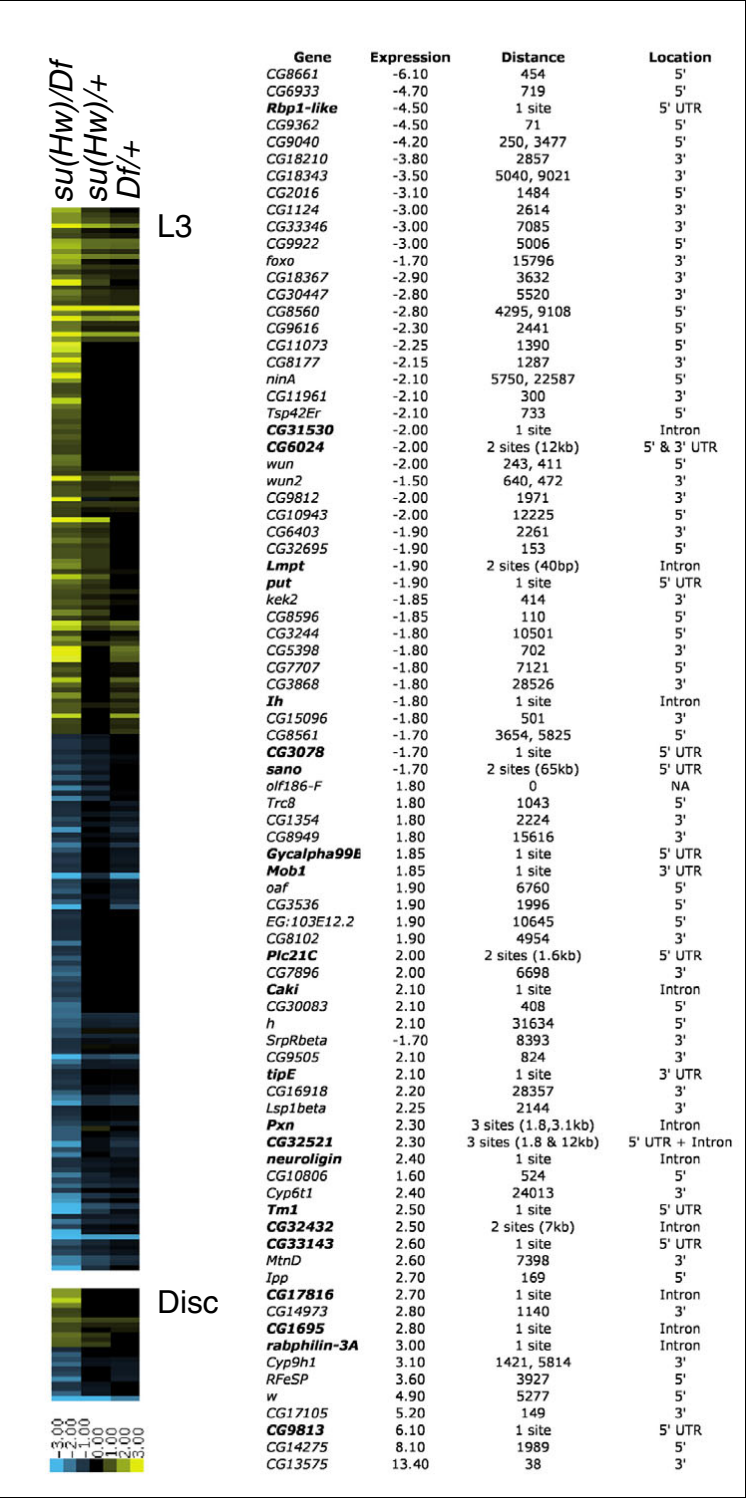
Genes with expression changes in su(Hw) mutant larvae (L3) and wing discs. A cluster diagram showing changes in gene expression in a *su*(*Hw*) null condition compared with changes in the heterozygous controls (fold change ≥ 1.7, *P *≤ 10^-3 ^for the mutants and approximately half the fold change at *P *≤ 10^-2 ^for the heterozygotes). The table lists those genes with greater than 1.7-fold expression change that have predicted Suppressor of Hairy-wing (Su [Hw]) binding sites within 30 kilobases (kb). The Expression column shows the absolute fold change for each gene. The Distance column indicates the distance between the gene model and Su(Hw) sites(s); for those genes with predicted sites within the gene model, the number of sites are indicated. If there is more than one site, the distance between them is given. The Location column indicates where the predicted sites lie with respect to the gene models. UTR, untranslated region.

Summarizing the expression data, in the whole animal we found 838 genes with greater than 1.7-fold expression change in the *su*(*Hw*) null compared with wild-type (*P *≤ 10^-2^). Restricting this to a more conservative *P *value cut-off of ≤10^-3^, we detect 405 genes with greater than a 1.7-fold change. Filtering this list to remove genes that also showed changes in the two control heterozygous conditions, eliminating genes with a fold change approximately half or more of that in the homozygous condition and a *P *value ≤ 10^-2^, left 206 genes (Figure 9 and Additional data file 5). In the case of the wing disc, 89 genes showed a greater than 1.7-fold change (*P *≤ 10^-2^), 37 changed at the more stringent *P *value (≤10^-3^), and 22 remained after filtering changes in the control heterozygotes (Figure 9 and Additional data file 6). The filtered lists overlap by nine genes: *activin-beta*, *B52*, *CG5590*, *CG9027*, *CG9362, CG9813*, *eIF-4E*, *ImpL2*, and *su*(*Hw*). We conducted an analysis to look for any over-represented features in the set of differentially expressed genes (Gene Ontology annotation, chromosomal position, clustering, or presence of introns) but found no significant associations. Focusing on the *Adh *region, we relaxed our selection criteria and from the 229 genes represented on the array identified 19 genes from whole larvae and three genes from wing discs with more than 1.4-fold change (*P *≤ 10^-2^), with a single gene (*CG4930*) common to both datasets (Figure 7 and Additional data files 7 and 8).

We looked at the association between genes with changed expression and predicted *in vivo *Su(Hw) binding sites. At a genome-wide scale we identified 83 genes with a 1.5-fold or greater change in expression (*P *≤ 10^-2^) that have a predicted Su(Hw) binding site within 30 kb (Figure 9). Of these, 24 genes have predicted binding sites within the gene model and seven of these genes have more than one site; none of the sites are in predicted coding sequence. We identified five cases in which adjacent genes, separated by a Su(Hw) binding site, both show expression changes in *su*(*Hw*) null mutants. In four of these cases the adjacent genes are divergently transcribed (*CG2016 *and *CG1124*, *CG9922 *and *foxo*, *wun *and *wun2*, and *CG10806 *and *neuroligin*) and in the remaining case they are convergently transcribed (*SrpRbeta *and *h*). With two of these paired genes, the intergenic region contains two Su(Hw) sites. Again focusing on the *Adh *region, for which we have ChIP binding data, we looked for an association between Su(Hw) binding site clustering and changes in gene expression but found none (Figure 7). Taken the findings together, we draw the following conclusions: loss of *su*(*Hw*) has widespread general effects on gene expression; many changes in gene expression are not associated with closely spaced Su(Hw) binding sites; and of those genes that show altered expression in *su*(*Hw*) mutants and that have at least one associated Su(Hw) site, the majority have only a single site.

## Discussion

Using ChIP array we have identified approximately 60 sites across the 3 Mb *Adh *genomic region that are bound by Su(Hw) *in vivo *(Figure 1), representing a large increase in the number of identified Su(Hw) binding sites. Analysis of these endogenous Su(Hw) binding sites allowed considerable expansion of the Su(Hw) consensus binding sequence. The existing Su(Hw) binding consensus was formed from the 12 sites in the 5'-untranslated region of the *gypsy *transposable element. These sites provided a consensus 12 bp sequence, 5'(TC)(AG)(TC)TGCATA(CT)(TC)(TC), separated by short, variable AT-rich sequences. As shown in Figure 3, the Su(Hw) consensus derived for the endogenous sites shows sequence preference extending over 20 bp that fits very well with the region of DNA-protein interaction defined by Spana and Corces [32]. This long consensus also fits with the 12 zinc finger domain structure of Su(Hw) and with the striking observation that a high scoring consensus match is highly predictive of protein binding *in vivo *(Figures 1 and 4). This latter finding strongly contrasts with the general experience of transcription factor binding site analysis, in which commonly only a small proportion of the binding sites predicted by sequence are found to be occupied *in vivo*. This was observed, for example, in the ChIP-array analyses of yeast transcription factors [39,40] and lies at the heart of the difficulty in predicting transcription factor targets by *in silico *analysis.

The Su(Hw) results presented here can be contrasted with our previously reported analysis of the genomic binding sites for the heat shock transcription factor Hsf. Even if we only consider perfect matches to the consensus Hsf binding site, GAANNTTCNNGAA, this gives a minimum number of 32 sites across the 3 Mb *Adh *region, whereas ChIP array analysis indicates clear *in vivo *Hsf occupancy at only two sites [26]. Considering that many functional Hsf binding sites are less-than-perfect matches to the consensus, this indicates that only a very small fraction of potential Hsf binding sites are actually occupied *in vivo*. There may be several explanations for why matches to consensus binding sites are not good predictors of *in vivo *occupancy; for example, the consensus sites may be poorly characterized or the binding of transcription factors may often involve a particular context and neighbouring co-factor binding may be required. Alternatively, many potential binding sites may be obscured by other DNA-binding proteins, by histones or by higher order chromatin structure.

Our observation that high scoring matches to the consensus Su(Hw) site are good predictors of occupancy indicates that Su(Hw) may in some way be special. It may reflect the possibility that Su(Hw) binds on its own whereas many transcription factors achieve specificity through interactions with co-factors. In support of this conclusion, we did not find strong sequence conservation immediately flanking the Su(Hw) binding site; also, in the conservation that we observed by unbiased pattern matching in the MEME analysis, the highly conserved residues fit excellently with the contact residues previously described for Su(Hw) [32]. It can be speculated that the comparatively long Su(Hw) motif would functionally resemble a series of multiple shorter transcription factor binding sites. A direct connection between DNA sequence and Su(Hw) binding would also fit with the proposed chromosomal architectural role for Su(Hw) and may indicate that chromatin structure does not restrict the availability of Su(Hw) sites. A straightforward link between DNA sequence and Su(Hw) occupancy is also supported by the striking observation that the same set of binding sites is occupied by Su(Hw) in a variety of developmental stages and tissues. Our analysis of Su(Hw) binding site occupancy in 0 to 20 hour embryos, third instar larval brain, and third instar larval imaginal discs indicates that most sites, if not all, are constitutively occupied by Su(Hw). This lack of developmental regulation of Su(Hw) binding is consistent with a constant chromosomal architectural role for the Su(Hw) protein.

The presence of the AT tracts flanking the core Su(Hw) binding site suggested to Spana and Corces [32] that DNA bending may be involved in the interaction of Su(Hw) with its binding site. They tested this by mutating the flanking regions and concluded that DNA bending was a factor both for the binding of Su(Hw) *in vitro *and for *in vivo *insulator function. Interestingly, although we find that the endogenous sites we identified do not contain the same configuration of core and conserved A/T-rich flanking sequences, these sites nevertheless exhibit a strong bendability profile (Figure 8). This supports the idea that the local DNA conformation may play an important role in Su(Hw) target specificity.

A significant observation from our genomic analysis of Su(Hw) sites is that, in contrast to the 12 sites within the 340 bp *gypsy *insulator, endogenous sites are not arranged in clusters. This is in agreement with the characterization of the first endogenous site between *yellow *and *achaete*, in which the functional insulator only contains two putative Su(Hw) binding sites separated by 49 bp [20,21]. Indeed, in this case it is not entirely clear that there are two closely spaced *in vivo *binding sites. Although two Su(Hw) binding sites were capable of being band-shifted by Su(Hw) protein, only one molecule of Su(Hw) appears to be associated with a 125 bp fragment that contains both sites. We note that both sites score moderately well with our *in vivo *consensus (site 1A-1 Patser *P *= e^-13.5^, 1A-2 Patser *P *= e^-14.3^), although 1A-1 lacks the conserved G at position 17. Recent genome-wide analyses of matches to the *gypsy *Su(Hw) consensus also failed to find evidence of extensive site clustering [22,23]. The observation that our newly identified sequences exhibit a different bendability profile from the *gypsy *sequences may explain why multiple *gypsy *sequences are required for insulator activity, whereas the endogenous sites appear to function as single binding sites.

In this analysis we have found that the endogenous Su(Hw) sites differ in several ways from their counterparts in the *gypsy *insulator; they are not tightly clustered, they do not conform to the model of binding site core with flanking AT-rich elements, and they have a different DNA flexibility profile. What are the implications of these differences for the putative endogenous insulator function of these sequences? We can address the clustering issue. From their analysis of synthetic multimers of *gypsy *Su(Hw) binding sites, Scott and coworkers [31] found that four copies of the binding site were required for insulator function in a transgenic enhancer-blocking assay. This suggested that endogenous sites with insulator activity would also have clusters of binding sites, but the endogenous site between *yellow *and *achaete *has been demonstrated to have insulator activity despite having only the two *in vitro *Su(Hw) binding sites. More recently, Ramos and coworkers [22] used an *in vitro *pull down assay to identify a number of putative endogenous Su(Hw) binding sites, and they demonstrated insulator activity for two fragments, each containing only a single Su(Hw) site. Similar conclusions were reached for sites identified by *in silico *analysis [23]. Thus, there is good evidence that single Su(Hw) binding sites can mediate insulator function, and this suggests that either the endogenous sites are more potent than individual binding sites from the *gypsy *element (the *gypsy *sites do not score particularly highly against the *in vivo *consensus; the highest score has a *P *value of e^-13.9 ^and only two sites score better than *P *= e^-10^) or that the units used in the construction of the synthetic multiple site may not have contained all relevant sequences.

Mapping of the Su(Hw) binding sites to the annotated genome does not reveal an exclusive genomic niche for these sites; although most sites are intergenic, there are still many sites (37%) that overlap transcription units. Although this distribution of sites does not immediately lend support to a model in which Su(Hw) functions to partition the genome into separate regulatory domains, it is currently not clear what conclusions we can draw from this analysis of genomic environment. Apart from transcription units, there are at present few genomic features whose distribution we can compare with Su(Hw) sites. We have looked at the mapping of GAGA factor [41] and of the boundaries of neighbourhoods of co-regulated genes [42], but have not identified any revealing correlations.

If the 3 Mb *Adh *region is representative of the genome, then finding 60 sites in this region would predict over 2,000 sites across the *Drosophila *genome. Indeed, searching the whole genome using Patser (matches to consensus with *P *value < e^-15^) yields 2,282 sites, a figure that closely agrees with the 2,500 sites predicted by Ramos and coworkers [22]. How does this relate to the several hundred sites (between 200 and 500) observed in immunolabeling studies on polytene chromosomes [10,19]? This difference could simply reflect the level of resolution of the analysis. However, the comparison between several hundred bands in polytene chromosomes and only 20 to 50 Su(Hw) nuclear puncta in diploid cells has been interpreted to mean that the nuclear puncta represent aggregations of Su(Hw) binding sites [19]. If this argument is also applicable to polytene chromosomes, then the observation of several hundred bands representing more than 2,000 binding sites might indicate that the Su(Hw) binding sites can associate together within the structure of the polytene chromosomes.

From a technical perspective, we have demonstrated the feasibility of ChIP-array analysis with small amounts of specific tissues prepared by dissection. This provides a basis for developmental analysis that allows correlation of binding site occupancy with the progression of cell fate decisions during development. Also, the success of the GFP tagged approach provides an alternative strategy for the general mapping of the binding sites of chromatin associated proteins in *Drosophila*; a GFP gene-trap strategy may be preferable to the prospect of producing specific antibodies against all the chromatin associated proteins in *Drosophila*.

To explore Su(Hw) function we investigated the transcriptional consequences of lack of Su(Hw) using genome-wide microarray analysis. Examining gene expression changes in *su*(*Hw*) null mutant larvae and wing discs, we find widespread changes in gene expression with consistent upregulation and downregulation of many genes. There are no obvious features (gene structure or Gene Ontology classification) that relate the genes with altered expression. Rather, it appears that the effects of loss of Su(Hw) are general. Importantly, fewer than 20% of genes with expression changes are associated with a Su(Hw) binding site within 1 kb of the transcription unit, and of these genes only three are associated with two sites within 1 kb of each other. This supports the contention that endogenous Su(Hw) function is not mediated through clustered binding sites. We recognize that some of the transcriptional changes we observe may reflect compensatory alterations in gene expression in response to loss of Su(Hw) earlier in development. A more detailed analysis of the transcriptional response to loss of Su(Hw) in specific tissues, focusing on the immediate results of removing Su(Hw), will be required to define clearly the role played by Su(Hw) in regulating particular genes. Nevertheless, the reproducible RNA expression changes in *su*(*Hw*) mutants represent a clear molecular phenotype. Although the changes are reproducible and significant, they do not result in a visible phenotype, and the only clear phenotype in *su*(*Hw*) mutants is female sterility. Given the widespread gene expression changes we observe in larvae and imaginal discs, it appears likely that this phenotype represents a specific sensitivity of oogenesis to changes in gene expression rather than a specific requirement for Su(Hw) in this tissue.

## Conclusion

By mapping binding sites of the insulator protein Su(Hw), we have provided a genomic framework for the analysis of the endogenous Su(Hw) function. We find that genomic binding sites for Su(Hw) generally occur as isolated single sites. The high degree of conservation of these sites and the widespread transcriptional effects of loss of Su(Hw) indicate a role for these dispersed sites in transcriptional regulation and fit with a proposed general role of Su(Hw) in the regulatory architecture of the genome.

## Materials and methods

### Fly strains and antibodies

The wild-type strain used was OregonR. A Su(Hw)-GFP transgenic line containing a GFP-tagged version of *su*(*Hw*), *y*[2]; *P*{*su*(*Hw*).*GFP*}; *Df*(*3R*)*su*(*Hw*)^*V*^, *P{casX/K-RpII15} *[17] and rabbit and rat anti-Su(Hw) antisera were obtained from Victor Corces (Johns Hopkins University, Baltimore MD, USA). The affinity-purified rabbit anti-GFP was generated by Palacios and coworkers [43]. For expression profiling, we used the *su*(*Hw*) null strain *su*(*Hw*)^*V*^, *P [CaS X/K5.3] *[17], in combination with the DrosDel deletion *Df*(*3R*)*ED5644 *(a 600 kb deletion encompassing 84A4-88C9 [44]) to produce a null background. The *su*(*Hw*)^*V*^, *P [CaS X/K5.3] *chromosome is poorly viable when homozygous, presumably because of the accumulation of deleterious mutations elsewhere on the chromosome. However, it is healthy over other hypomorphic *su*(*Hw*) alleles and in combination with the *ED5644 *deletion. To obtain *su*(*Hw*) null animals we crossed *su*(*Hw*)^*V*^, *P *[*CaS X*/*K5.3*]/*TM6B*, *Tb *with *Df*(*3R*)*ED5644*/*TM6B*, *Tb *and recovered nontubby third instar larvae. Heterozygotes for each of the *su*(*Hw*) mutant chromosomes were recovered by selecting the nontubby larvae from outcrosses to a wild-type Oregon-R stock. To ensure that animals were precisely age matched, larvae were selected at the soft white pre-pupal stage just after everting their anterior spiracles [45].

### Chromatin isolation and immunopurification

Chromatin from embryos aged between 0 and 20 hours after egg laying was purified as described previously [26]. For the preparation of chromatin from brain and wing discs, late third instar larvae were dissected in ice-cold Schneider's medium. Dissected brains and discs were washed with phosphate-buffered saline, fixed in phosphate-buffered saline/1.5% formaldehyde for 20 min, and washed with phosphate-buffered saline. Batches of material were snap-frozen in liquid nitrogen and stored at -80°C. Chromatin was prepared from a minimum of 20 brains or 50 discs. For immunopurification, in a 300 μl reaction, the specific immunopurification used 2.5 μl rat anti-Su(Hw) antiserum followed by 1 μg rabbit anti-rat Ig (Jackson ImmunoResearch Laboratories, West Grove PA, USA) or 0.06 to 0.1 μg affinity-purified rabbit anti-GFP; the control immunopurification used either 1 μg rabbit anti-β-galactosidase (Rockland, Gilbertsville PA, USA) or 1 μl normal rabbit antiserum. ChIP enrichment was assayed using PCR with specific primers as described previously [26]. The primers used were as follows: gypsy, tcaaaaaataagtgctgcatacttttt and gagcacaattgatgcgcta; 1A-2, tccacctgctactatcccta and ccctgattacacaacaaggt; and *Gpdh*, acgctgacatcctgatcttc and atagaagacgtccacgaagc.

### Genomic microarray analysis

The array description is available from Gene Expression Omnibus under platform accession GPL3689 [29]. Amplification and labeling of DNA from enriched chromatin, as well as hybridizations to genomic DNA tiling arrays, were performed as described previously [26]. For the experiment comparing anti-Su(Hw) with anti-GFP, in each case two independent biological chromatin preparations were used for the specific immunopurifications, along with parallel control immunopurifications. Each labeling was technically replicated via a dye swap, giving a total of four hybridizations for each antibody. In the case of the remaining anti-GFP immunopurifications, four independent chromatin biological replicates were prepared from each tissue source. With the embryo chromatin, each of the biological replicates was technically replicated via a dye-swap, generating eight slides. In the case of the brain and wing tissue, three of the biological replicates were technically replicated via a dye-swap, giving six slides. The remaining immunopurification for each tissue was independently amplified twice, and each of these technically replicated via a dye swap, giving a further four slides. Thus, a total of ten microarray hybridizations were performed for each of the tissues, but one of the wing slides failed, giving nine slides in this case. Microarray scanning, spot finding, and VSN normalization were performed as described by Birch-Machin and coworkers [26] and on the FlyChip web site [46]. For each biological replicate the ratios for the technical replicates were averaged and statistical significance across biological replicates assessed using the CyberT framework [28,47].

### Motif finding, data depiction, and further data analysis

All other data analyses were performed using custom-written Perl scripts or publicly available websites. Motif finding was carried out using the Motif Discovery tool on the Multiple EM for Motif Elicitation v3.0 website [30,48]. Parameters were optimized to discover up to six motifs between 10 and 20 nucleotides in length. The site stack for the *bona fide *Su(Hw) binding motif was then used to create a position-specific weight matrix (Additional data file 2) for the Patser Web interface [49]. Sequences of microarray DNA fragments were searched against this position-specific weight matrix, and relative distances of the Patser hits in respect to the fragment length were transposed to coordinates of the *D. melanogaster *genome sequence (release 4). The consensus sequence for the Su(Hw) binding motif was depicted using the MEME site stack in WebLogo [50,51].

The binding site profile across the *Adh *region was created using Gaussian smoothing of the enrichment factors across 10 (+5/-5) neighboring fragments. Data on the evolutionary conservation of potential Su(Hw) binding sites were obtained by comparison of predicted Patser sites with the 'PhastCons' multiple alignment data available from the University of California, Santa Cruz (UCSC) Genome Browser Web site [52]. Patser hits within fragments that showed enrichment in the microarray experiments were correlated against genomic coordinates of genetic features of coding and noncoding Flybase genes (obtained from UCSC Genome Browser). Biophysical properties of the putative Su(Hw) binding sites were determined for the 100 best Patser hits to the consensus and their genomic context, 100 random fragments of the same length, and the 12 Su(Hw) binding sites in the *gypsy *insulator. Profiles of free stacking energy (coefficients from Protozanova and coworkers [38]) were averaged for the three groups, following the strategy described by Liao and colleagues [53].

### Gene expression profiling of Su(Hw) mutants

Gene expression analysis was carried out using the FL002 (INDAC) whole transcriptome long oligonucleotide microarrays developed and printed in the FlyChip microarray facility [46]. RNA was prepared from larvae and wing discs using standard Trizol extraction, as described on the FlyChip website [46]. For whole larvae, RNA samples were labeled by direct incorporation of Cy3 or Cy5 dyes during first strand cDNA synthesis. For wing discs, RNA from ten pairs of discs was amplified using modifications to standard T7-polymerase protocols, as detailed on the FlyChip website [46]. For each comparison, four independent biological replicates were prepared and hybridized with a wild-type control sample to the arrays in a dye swap configuration (two Cy3 versus Cy5, and two Cy5 versus Cy3). Slides were scanned using an Axon 4000B (Molecular Devices Corporation, Union City CA, USA), and spot finding was performed using the Dapple software [54] and normalized using a custom implementation of the VSN method. Detailed protocols are available from the FlyChip website. All 12 whole larval slides were normalized together, as were the 12 wing disc slides. Average ratios and statistics were calculated with the CyberT package. Raw microarray data are available from the National Center for Biotechnology Information Gene Expression Omnibus site [29] (Platform ID GPL5135 [INDAC_CAM_FL002], series IDGSE7682: Gene expression in *su*(*Hw*) null larvae) and summarized in Additional data files 5 to 8.

## Abbreviations

bp, base pairs; ChIP, chromatin immunopurification; CTCF, CCCTC binding factor; GFP, green fluorescent protein; kb, kilobases; Mb, megabases; PCR, polymerase chain reaction; Su(Hw), Suppressor of Hairy-wing; UCSC, University of California, Santa Cruz; VSN, variance stabilization normalization.

## Authors' contributions

BA and GW performed the ChIP-array experiments and analysed the results. IB-M established the chromatin preparation and ChIP protocols. SG established the microarray platform. MQ performed validation ChIP experiments. LM performed the gene expression profiling. SR and RW conceived the experiments and participated in experimental design and analysis. BA, SR and RW wrote the manuscript. All authors read and approved the final version of the manuscript.

## Additional data files

The following additional data are available with the online version of this paper. Additional data file 1 is a table listing the ChIP-array results. Additional data file 2 contains the Su(Hw) Position Specific Weight matrix. Additional data file 3 is a table listing the Patser data. Additional data file 4 is a figure showing the validation ChIP assays. Additional data file 5 is a table listing gene expression changes in total third instar larvae. Additional data file 6 is a table listing gene expression changes in wing discs. Additional data file 7 is a table listing gene expression changes in total third instar larvae, showing CyberT results for genes in the *Adh *region. Additional data file 8 is a table listing gene expression changes in wing imaginal discs, showing CyberT results for genes in the *Adh *region. Additional data file 9 provides genome browser annotation tracks with coordinates in *Drosophila *genome, release 3.1.

## Supplementary Material

Additional data file 1 Provided is a table listing the ChIP-array results, showing the array ID number, fragment ID, and the values for the mean enrichment, *P *value, and *t*-value derived by CyberT from the embryo, brain, and wing ChIP-array data. Click here for file

Additional data file 2 Provided is a document containing the Su(Hw) position specific weight matrix. Click here for file

Additional data file 3 Provided is a table listing the Patser data, showing fragment ID and the associated Patser matches to the Su(Hw) position specific weight matrix, with details of strand, sequence of match, match Patser score, match Patser *P *value as ln(*P*), and chromosome and nucleotide coordinates in *Drosophila *genome release 3.1. Click here for file

Additional data file 4 Provided is a figure showing the validation ChIP assays to test prediction of binding sites outside the *Adh *region. We selected eight sites across the genome with good matches to the weight matrix (Patser *P *values ranged from e^-16.3 ^to e^-21.5^) and that did not match the consensus (CT)(AT)GC(AC)TACTT(ACT)(CT) presented by Ramos and coworkers [22]. The specific immunopurification used 1 μl rabbit anti-Su(Hw) and the control used 1 μl normal rabbit serum, each in a 300 μl reaction using chromatin from *Drosophila *embryos. ChIP enrichment was assayed using PCR with specific primers, as described previously [26]. Six out of the eight test sites (sites 1 to 6) exhibited clear enrichment, and a further site site (site 7) exhibited weak evidence for enrichment. The primers used were as follows: 1A-2 (see Materials and Methods); 1, tttgctcaatgcaaagcact and gaatgaactgccgtccaact; 2, ccgatcctgcaagagaaaaa and tcaaccgagtacgagtgtgc; 3, ggcctaccgcaaaattcat and gggcaactcattaggcagtc; 4, tgctgtttcttcgagggagt and atgctttggttgcccattac; 5, catgtacgatctgcggaatg and cgcactccaagtgaagaaca; 6, caacattcgccattgcatac and ccacaaatccgctttcaaat; 7, caggccaaaaggcagttcta and tcagagattcgtggcagttg; and 8, cacactcgaagcgtgtgaat and aagtgtgtttgccagtgtgc. Click here for file

Additional data file 5 Provided is a table listing gene expression changes in total third instar larvae, showing CyberT results for 838 genes with better than 1.7-fold (*P *≤ 10^-2^) in the following: su(Hw)/Df (*su*(*Hw*)^*v*^, *P *[*CaS X*/*K5.3*]/*Df*(*3R*)*ED5644*), *Su*(*Hw*)/+(*su*(*Hw*)^*v*^, *P *[*CaS X*/*K5.3*]/+), and *Df*/+ (*Df*(*3R*)*ED5644*/+). FlyBaseID, Flybase gene identifier; FlyBAse_sym = gene symbol; GeneID, FlyBase annotation symbol; ID, array unique identifier; Mn, mean log ratio of replicates; #obs, slides passing QC filters; p = *P *value; SD, standard deviation; t, t-statistic. Click here for file

Additional data file 6 Presented is a table listing gene expression changes in wing discs showing CyberT results for 89 genes with better than 1.7-fold (*P *≤ 10^-2^) in the following: su(Hw)/Df (*su*(*Hw*)^*v*^, *P *[*CaS X*/*K5.3*]/*Df*(*3R*)*ED5644*), *Su*(*Hw*)/+ (*su*(*Hw*)^*v*^, *P *[*CaS X*/*K5.3*]/+), and *Df*/+ (*Df*(*3R*)*ED5644*/+). Column headings are as for Additional data file 5. Click here for file

Additional data file 7 Presented is a table listing gene expression changes in total third instar larvae showing CyberT results for the 229 genes in the *Adh *region: su(Hw)/Df (*su*(*Hw*)^*v*^, *P *[*CaS X*/*K5.3*]/*Df*(*3R*)*ED5644*), *Su*(*Hw*)/+ (*su*(*Hw*)^*v*^, *P *[*CaS X*/*K5.3*]/+), and *Df*/+ (*Df*(*3R*)*ED5644*/+). Column headings are as for Additional data file 5. Click here for file

Additional data file 8 Presented is a table listing gene expression changes in wing imaginal discs showing CyberT results for the 229 genes in the *Adh *region: su(Hw)/Df (*su*(*Hw*)^*v*^, *P *[*CaS X*/*K5.3*]/*Df*(*3R*)*ED5644*), *Su*(*Hw*)/+ (*su*(*Hw*)^*v*^, *P *[*CaS X*/*K5.3*]/+), and *Df*/+ (*Df*(*3R*)*ED5644*/+). Column headings are as for Additional data file 5. Click here for file

Additional data file 9 Provided are genome browser annotation tracks with coordinates in *Drosophila *genome, release 3.1. Click here for file
