# Supplementary figures and images for: Genomic mapping of Suppressor of Hairy-wing binding sites in Drosophila
Source: Genome Biol. 2007 Aug 16;8(8):R167. doi: 10.1186/gb-2007-8-8-r167 (PMC2374998; doi:10.1186/gb-2007-8-8-r167)

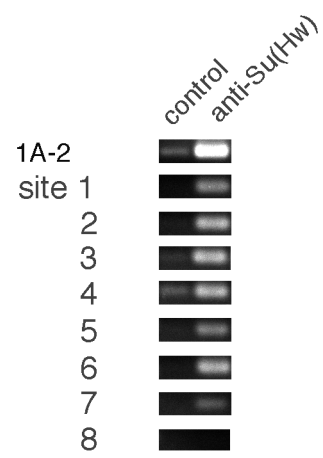

Supplement: Additional data file 4 — Provided is a figure showing the validation ChIP assays to test prediction of binding sites outside the Adh region. We selected eight sites across the genome with good matches to the weight matrix (Patser P values ranged from e-16.3 to e-21.5) and that did not match the consensus (CT)(AT)GC(AC)TACTT(ACT)(CT) presented by Ramos and coworkers [22]. The specific immunopurification used 1 μl rabbit anti-Su(Hw) and the control used 1 μl normal rabbit serum, each in a 300 μl reaction using chromatin from Drosophila embryos. ChIP enrichment was assayed using PCR with specific primers, as described previously [26]. Six out of the eight test sites (sites 1 to 6) exhibited clear enrichment, and a further site site (site 7) exhibited weak evidence for enrichment. The primers used were as follows: 1A-2 (see Materials and Methods); 1, tttgctcaatgcaaagcact and gaatgaactgccgtccaact; 2, ccgatcctgcaagagaaaaa and tcaaccgagtacgagtgtgc; 3, ggcctaccgcaaaattcat and gggcaactcattaggcagtc; 4, tgctgtttcttcgagggagt and atgctttggttgcccattac; 5, catgtacgatctgcggaatg and cgcactccaagtgaagaaca; 6, caacattcgccattgcatac and ccacaaatccgctttcaaat; 7, caggccaaaaggcagttcta and tcagagattcgtggcagttg; and 8, cacactcgaagcgtgtgaat and aagtgtgtttgccagtgtgc. [file gb-2007-8-8-r167-S4.pdf]
