# Supplementary material for: Genomic mapping of Suppressor of Hairy-wing binding sites in Drosophila
Source: Genome Biol. 2007 Aug 16;8(8):R167. doi: 10.1186/gb-2007-8-8-r167 (PMC2374998; doi:10.1186/gb-2007-8-8-r167)
Supplement: Additional data file 9 — Provided are genome browser annotation tracks with coordinates in Drosophila genome, release 3.1. [file gb-2007-8-8-r167-S9.pdf]

Annotation Tracks for Genome Browser

Su(Hw)1: *Adh* region sites that match the consensus with a p value of –12 or better and that map to fragments with enrichment of at least 1.7 fold in ChIP from at least one of the chromatin sources. 60 sites

Su(Hw)2: *Adh* region sites that match the consensus with a p value between –12 and –15 and that map to fragments with enrichment of at least 1.7 fold in ChIP from at least one of the chromatin sources. 18 sites.

Su(Hw)3: *Adh* region sites that match the consensus with a p value of –15 or better and that map to fragments with enrichment of at least 1.7 fold in ChIP from at least one of the chromatin sources. 42 sites.

Su(Hw)4: *Adh* region sites that match the consensus with a p value of –15 or better and that map to fragments whose ChIP enrichment does not pass the 1.7 fold threshold. 37 sites.

```
--------------------------- cut below and paste for UCSC ------------------------
browser position chr2L:13500000-16500000
track name=Su(Hw)1 description="SuHw Patser p value <= -12 and enrichment >= 1.7 fold" color=255,0,0
chr2L	13584993	13585012
chr2L	13607659	13607678
chr2L	13614204	13614223
chr2L	13629839	13629858
chr2L	13632623	13632642
chr2L	13638889	13638908
chr2L	13641670	13641689
chr2L	13667151	13667170
chr2L	13677997	13678016
chr2L	13714589	13714608
chr2L	13824516	13824535
chr2L	13894059	13894078
chr2L	13942758	13942777
chr2L	13969073	13969092
chr2L	13973053	13973072
chr2L	13988200	13988219
chr2L	14014963	14014982
chr2L	14020712	14020731
chr2L	14041051	14041070
chr2L	14082802	14082821
chr2L	14089608	14089627
chr2L	14134128	14134147
chr2L	14134237	14134256
chr2L	14166557	14166576
chr2L	14175641	14175660
chr2L	14179505	14179524
```

chr2L 14179577 14179596
chr2L 14234119 14234138
chr2L 14234544 14234563
chr2L 14555301 14555320
chr2L 14601019 14601038
chr2L 14688545 14688564
chr2L 14807926 14807945
chr2L 14864561 14864580
chr2L 14919464 14919483
chr2L 15052715 15052734
chr2L 15053529 15053548
chr2L 15075047 15075066
chr2L 15137627 15137646
chr2L 15159546 15159565
chr2L 15555313 15555332
chr2L 15591842 15591861
chr2L 15631187 15631206
chr2L 15634144 15634163
chr2L 15634525 15634544
chr2L 15660497 15660516
chr2L 15667741 15667760
chr2L 15668122 15668141
chr2L 15695494 15695513
chr2L 15717602 15717621
chr2L 15774643 15774662
chr2L 15811071 15811090
chr2L 15898198 15898217
chr2L 15910924 15910943
chr2L 15946636 15946655
chr2L 16116870 16116889
chr2L 16349908 16349927
chr2L 16352558 16352577
chr2L 16355644 16355663
chr2L 16402841 16402860
track name=Su(Hw)2 description=" SuHw –15< Patser p value <= -12 and enrichment >= 1.7 fold " color=0,0,255
chr2L 13973053 13973072
chr2L 14134237 14134256
chr2L 14175641 14175660
chr2L 14179505 14179524
chr2L 14179577 14179596
chr2L 14601019 14601038
chr2L 15052715 15052734
chr2L 15591842 15591861
chr2L 15631187 15631206
chr2L 15634144 15634163
chr2L 15634525 15634544
chr2L 15667741 15667760
chr2L 15668122 15668141
chr2L 15774643 15774662

chr2L 15811071 15811090
chr2L 15946636 15946655
chr2L 16349908 16349927
chr2L 16402841 16402860
track name=Su(Hw)3 description=" SuHw Patser p value <= -15 and enrichment >= 1.7 fold " color=255,0,0
chr2L 13584993 13585012
chr2L 13607659 13607678
chr2L 13614204 13614223
chr2L 13629839 13629858
chr2L 13632623 13632642
chr2L 13638889 13638908
chr2L 13641670 13641689
chr2L 13667151 13667170
chr2L 13677997 13678016
chr2L 13714589 13714608
chr2L 13824516 13824535
chr2L 13894059 13894078
chr2L 13942758 13942777
chr2L 13969073 13969092
chr2L 13988200 13988219
chr2L 14014963 14014982
chr2L 14020712 14020731
chr2L 14041051 14041070
chr2L 14082802 14082821
chr2L 14089608 14089627
chr2L 14134128 14134147
chr2L 14166557 14166576
chr2L 14234119 14234138
chr2L 14234544 14234563
chr2L 14555301 14555320
chr2L 14688545 14688564
chr2L 14807926 14807945
chr2L 14864561 14864580
chr2L 14919464 14919483
chr2L 15053529 15053548
chr2L 15075047 15075066
chr2L 15137627 15137646
chr2L 15159546 15159565
chr2L 15555313 15555332
chr2L 15660497 15660516
chr2L 15695494 15695513
chr2L 15717602 15717621
chr2L 15898198 15898217
chr2L 15910924 15910943
chr2L 16116870 16116889
chr2L 16352558 16352577
chr2L 16355644 16355663
track name=Su(Hw)4 description=" SuHw Patser p value < -15 and enrichment < 1.7 fold " color=128,128,128

chr2L	13576244	13576263
chr2L	13632623	13632642
chr2L	13641670	13641689
chr2L	13749739	13749758
chr2L	13969073	13969092
chr2L	14057173	14057192
chr2L	14106974	14106993
chr2L	14110653	14110672
chr2L	14352281	14352300
chr2L	14688545	14688564
chr2L	14788814	14788833
chr2L	14882942	14882961
chr2L	14939553	14939572
chr2L	14991308	14991327
chr2L	14998235	14998254
chr2L	15056641	15056660
chr2L	15137627	15137646
chr2L	15159546	15159565
chr2L	15212509	15212528
chr2L	15213622	15213641
chr2L	15281950	15281969
chr2L	15370457	15370476
chr2L	15543249	15543268
chr2L	15615023	15615042
chr2L	15658912	15658931
chr2L	15693909	15693928
chr2L	15836263	15836282
chr2L	15872750	15872769
chr2L	15932483	15932502
chr2L	15952587	15952606
chr2L	15961036	15961055
chr2L	16013343	16013362
chr2L	16042590	16042609
chr2L	16056445	16056464
chr2L	16129490	16129509
chr2L	16327530	16327549
chr2L	16365484	16365503
