# Supplementary material for: Genomic mapping of Suppressor of Hairy-wing binding sites in Drosophila
Source: Genome Biol. 2007 Aug 16;8(8):R167. doi: 10.1186/gb-2007-8-8-r167 (PMC2374998; doi:10.1186/gb-2007-8-8-r167)
Supplement: Additional data file 2 — Provided is a document containing the Su(Hw) position specific weight matrix. [file gb-2007-8-8-r167-S2.pdf]

Su(Hw) position-specific weight matrix

width of the alignment matrix: 20

A	8	2	14	0	0	32	0	40	4	4	0	3	3	9	2	0	4	6	3	14
C	0	5	0	0	40	10	1	0	37	0	1	6	8	9	3	2	20	0	20	17
G	32	2	5	42	0	0	0	2	1	0	0	0	0	17	30	40	15	26	11	2
T	2	33	23	0	2	0	41	0	0	38	41	33	31	7	7	0	3	10	8	9
